# The impact of sarcopenia and frailty on decompensation in compensated cirrhosis: A systematic review

**DOI:** 10.1097/HC9.0000000000000811

**Published:** 2025-10-21

**Authors:** Chiara Becchetti, Elton Dajti, Grace L. Su, Christian Labenz, Antonio Colecchia, Sith Siramolpiwat, Yoji Ishizu, Kathleen P. Ismond, Lucian Beer, Rafael Paternostro, Simone Di Cola, Jia Luo, Puneeta Tandon, Elliot B. Tapper, Ahmed Ba-Ssalamah, Thomas Reiberger, Manuela Merli, Jidong Jia, Annalisa Berzigotti, Cristina Ripoll, Susana G. Rodrigues

**Affiliations:** 1 Department for Visceral Surgery and Medicine, Inselspital, Bern University Hospital, University of Bern, Bern, Switzerland; 2 Graduate School for Health Sciences, University of Bern, Bern, Switzerland; 3 Hepatology and Gastroenterology Division, ASST Grande Ospedale Metropolitano Niguarda, Niguarda Hospital, Milan, Italy; 4 Gastroenterology Unit, IRCCS Azienda Ospedaliero-Universitaria di Bologna, Bologna, Italy; 5 Division of Gastroenterology and Hepatology, University of Michigan, Ann Arbor, Michigan, USA; 6 Department of Internal Medicine I, University Medical Center of the Johannes Gutenberg-University, Mainz, Germany; 7 Gastroenterology Unit, University Hospital of Modena, Department of Medical Specialities, University of Modena e Reggio Emilia, Modena, Italy; 8 Division of Gastroenterology, Department of Internal Medicine, Faculty of Medicine, Thammasat University, Bangkok, Thailand; 9 Department of Gastroenterology and Hepatology, Nagoya University Hospital, Nagoya, Japan; 10 Division of Gastroenterology, Faculty of Medicine and Dentistry, University of Alberta, Edmonton, Alberta, Canada; 11 Division of General and Paediatric Radiology, Department of Biomedical Imaging and Image-guided Therapy, Medical University of Vienna, Vienna, Austria; 12 Division of Gastroenterology and Hepatology, Department of Medicine III, Medical University of Vienna, Vienna, Austria; 13 Department of Translational and Precision Medicine, Sapienza University of Rome, Rome, Italy; 14 Department of Geriatrics, Beijing Friendship Hospital, Capital Medical University, Xicheng District, Beijing, China; 15 Liver Research Center, Beijing Friendship Hospital, Beijing Key Laboratory of Translational Medicine on Liver Cirrhosis, National Clinical Research Center of Digestive Diseases, Capital Medical University, Beijing, China; 16 Internal Medicine IV, Jena University Hospital, Friedrich Schiller University, Jena, Germany

**Keywords:** ascites, body composition, frail, muscle wasting, prognosis

## Abstract

**Background:**

Sarcopenia and frailty have a negative prognostic impact in patients with decompensated cirrhosis; however, the impact of these conditions on the prognosis of compensated cirrhosis is unknown. We performed a systematic review to assess the effect of sarcopenia and frailty on decompensation in patients with compensated cirrhosis.

**Methods:**

We searched PubMed and Embase for English-language studies published up to April 2025. The primary outcome was decompensation and the secondary outcome was mortality. We included studies with available data on patients with compensated cirrhosis.

**Results:**

Eight studies reporting data on sarcopenia (n=829 patients) and 4 studies (n=552 patients) assessing frailty were included in this systematic review. The prevalence of sarcopenia varied from 8% to 63%. Computed tomography at the L3 level and liver frailty index were the methods most commonly used to evaluate sarcopenia and frailty, respectively. Sarcopenia in patients compensated at inclusion was associated with an increased risk of decompensation in some studies, but not in all. When selected patients with compensated cirrhosis and no previous decompensation were studied, most studies showed no increased risk for first decompensation within the follow-up (12–61 mo). Two studies out of the 4 reported a higher risk of decompensation and mortality in patients with frailty.

**Conclusions:**

We reported that sarcopenia was prevalent in patients with compensated cirrhosis, but, in most studies, did not lead to an independent, increased risk of first decompensation and death. Well-designed, prospective multicenter studies are essential to assess the association between sarcopenia, frailty, and the risk of first decompensation.

## Graphical abstract

**Figure:**
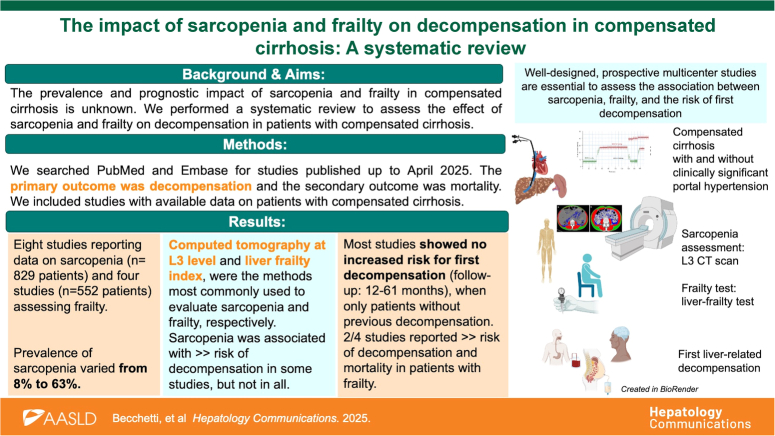

## INTRODUCTION

Sarcopenia is defined as reduced muscle mass, function, and quality, leading to negative effects on physical performance and clinical outcomes.^1^ Frailty is defined as increased vulnerability to health stressors^2^ and, specifically, physical frailty is seen as a parameter of reduced function and low muscle mass quality.^3^ Recent data support that both sarcopenia and frailty have a negative impact on the natural history of patients with decompensated advanced chronic liver disease (ACLD).^4^ This has been validated in patients with decompensated ACLD, particularly in the setting of liver transplantation (LT), where it is associated with higher morbidity and mortality.^5^^–^^7^

Despite the established association between sarcopenia and frailty and the risk of further decompensation and mortality in decompensated ACLD,^3^^,^^8^ their role in compensated cirrhosis remains unclear. Notably, the prevalence of sarcopenia and frailty in compensated cirrhosis is unknown, and whether they actively promote cirrhosis progression remains unclear. Furthermore, studies on compensated cirrhosis are scarce and often include mixed cohorts.^9^ Indeed, many studies define compensated cirrhosis by the current Child–Turcotte–Pugh stage and include patients who have had a previous episode of decompensation and have since clinically improved. This approach of not restricting patient selection to individuals who remain strictly compensated, that is, those who never experienced variceal bleeding, ascites, or HE, may be problematic, as the natural history is likely different from those with previous decompensation.^10^ In addition, several tools and cutoffs for defining sarcopenia and frailty have been used across studies, making comparisons challenging.

We performed a systematic review to assess the effect of sarcopenia and frailty on decompensation in patients with compensated cirrhosis.

## METHODS

### Search strategy

We conducted a systematic review in accordance with a pre-specified protocol registered in the PROSPERO repository (CRD42023414935). A comprehensive literature search was performed in PubMed and Embase to identify English-language articles published between 1st of January 2011, and 17th of 2025. According to our pre-registered protocol and following the recommendations of the Cochrane Handbook,^11^ we conducted a systematic review based on predefined eligibility criteria, independent screening of titles and abstracts by 2 reviewers, full-text assessment, data extraction using standardized forms, and quality appraisal of the included studies. Any disagreements were resolved through discussion or consultation with a third reviewer. We adopted the PRISMA 2020 guidelines^12^ (checklist enclosed in the Supplemental Material, http://links.lww.com/HC9/C124) to ensure transparent and complete reporting of the review process and findings. All research was conducted in accordance with both the Declarations of Helsinki and Istanbul. Given the design of the study, Institutional Review Board approval was waived.

The MeSH terms used were “sarcopenia” (and related terms: muscle mass loss, muscle mass wasting) AND “liver disease” OR “cirrhosis” for the first search, while for the second we used “frailty” (and related terms: muscle function) AND “liver disease” (and related terms: “cirrhosis”). The full search strategy is reported in the Supplemental Material, http://links.lww.com/HC9/C124.

Our search was restricted to human studies. During the first screening, we also considered reviews, conference abstracts, and letters to the editor. The screening was performed by 2 independent investigators (Chiara Becchetti and Susana G. Rodrigues). Discrepancies regarding inclusion were resolved through discussion. For studies with overlapping cohorts, we retained the most recent or largest sample sizes.

Eligible studies were included in the systematic review if they met the following criteria: (1) included patients with compensated cirrhosis based on clinical/histological/noninvasive methods, and consequently (2) had available data on decompensation as an outcome. Exclusion criteria were: (1) non-original studies; (2) non-target population (ie, only patients with decompensated cirrhosis, patients on the waiting list for LT, or xpatients with HCC); (3) studies with no data on follow-up or assessing clinical outcomes other than decompensation; and (4) studies with insufficient data and did not provide additional information on patients with compensated cirrhosis.

### Data extraction and quality assessment

The studies finally included were original studies, in which decompensation was considered among the outcomes. We used a standardized extraction form to extract the data for each included study. We selected studies in which the overall cohort included patients predominantly with compensated cirrhosis, as defined by the authors, which included at least one of the following: (1) clinical or histological signs of cirrhosis without a first liver-related decompensation, (2) MELD score <15, (3) Child–Pugh score A or B, and (4) clearly separated cohort of compensated and decompensated patients. In addition, the corresponding author of each study was contacted to retrieve the required missing data about decompensation and other data that were available, but not explicitly stated in the publication or Supplemental Material, http://links.lww.com/HC9/C124. Specifically, we retrieved information concerning the overall cohort, including anthropometric variables, etiology of liver disease, liver disease severity, data on sarcopenia, frailty and decompensation, overall decompensation rate, and overall survival rate. Furthermore, we contacted the authors regarding the definition of compensated cirrhosis and whether patients who had previous episodes of decompensation were included in this group. Subsequently, in the studies in which the author’s definition of compensated cirrhosis also included patients who were stable, but previously decompensated, we requested separate data according to the past occurrence of decompensation.

We separately analyzed studies referring to sarcopenia and frailty. We did not restrict our search to a specific methodology for defining sarcopenia or frailty, but collected information regarding each definition adopted. For decompensation, we included studies that considered decompensation events: ascites, variceal bleeding, and HE.^9^

The quality of the included studies was also scored by 2 independent investigators screening the studies (Chiara Becchetti and Susana G. Rodrigues) using the Newcastle–Ottawa scale (NOS)^13^ (Supplemental Material Table S1, http://links.lww.com/HC9/C124) with disagreements resolved after discussion.

### Statistical analysis

The primary outcome was the association between sarcopenia and frailty and the risk of decompensation, and the secondary outcome was mortality. For each study, we collected the cumulative incidence of the 2 clinical outcomes and the median follow-up time. Moreover, we evaluated how the multivariable model was built in terms of methodology (use of competing risk analysis or not) and variables included in the final model. We found significant heterogeneity in terms of outcome definition, type of analysis, and variables included in the multivariable model; therefore, no meta-analysis was performed.

## RESULTS

**FIGURE 1:**
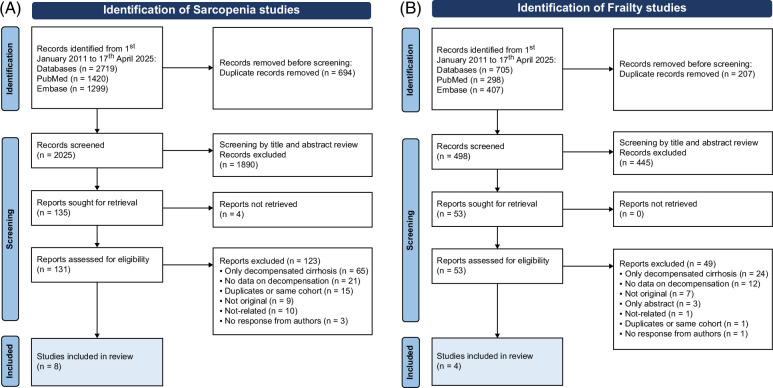
PRISMA flowchart of the studies included in the analysis for (A) sarcopenia and (B) frailty.

We identified 2719 records of sarcopenia and 705 records of frailty. After removing duplicates, we obtained 2025 for sarcopenia and 498 for sarcopenia and frailty, respectively. We refined our search by excluding 1890 and 445 items that did not match the inclusion criteria for sarcopenia and frailty, respectively. This large number of excluded studies was primarily due to cohorts of patients with mostly or exclusively decompensated cirrhosis, listed for LT, as well as patients with a high prevalence of HCC. Similarly, when decompensation was not considered among the outcomes or the cohorts were mixed (compensated and decompensated) and the authors could not provide separate data, these were excluded. As summarized in the PRISMA flowchart (Figure 1A), for sarcopenia we excluded 9 studies that were not original, 96 of which did not include the target population (ie, patients with HCC, liver transplant recipients, only decompensated), 15 of which were duplicated or considered the same cohort, and for 3 studies there was no reply from authors after 3 contacts. The same screening procedure was performed for frailty (Figure 1B). We excluded 7 studies that were not original, 37 that did not include the target population, and 5 in which data were considered insufficient. Overall, we included 8 studies on sarcopenia and 4 studies on frailty. All studies were considered to be of good quality (NOS score ≥7 points) (Supplemental Table S1, http://links.lww.com/HC9/C124).

### Sarcopenia

#### Characteristics of included studies and prevalence of sarcopenia

The general characteristics of the included studies are summarized in (Table 1, Supplemental Table S2, http://links.lww.com/HC9/C162), referring to the overall cohort provided as found in the published manuscript. Three of the 8 included studies were conducted outside Europe, specifically in the United States,^15^ Japan^17^ and China.^20^ Five studies were retrospective,^15^^–^^19^ and 3 were prospective.^14^^,^^20^^,^^21^ They all considered decompensation as clinically detected ascites, variceal bleeding, and HE leading to hospitalization. One study considered bleeding from portal hypertensive gastropathy^17^ as a decompensating event, another 2 studies considered also liver-related mortality^18^ and infection,^20^ respectively. No study has considered jaundice among the decompensation events.

Sarcopenia was diagnosed in all the studies using imaging criteria. In Beer et al^16^ MRI was used, while in all other studies, CT was the technique of choice. All measurements were performed at the L3 level, except for Tapper et al^15^ where T12 was adopted. The skeletal muscle index (SMI) was the most frequent type of measurement adopted (5/8 studies), and in all non-Asian studies, the cutoff proposed by Carey et al^24^ was used. In 2 studies,^16^^,^^18^ transversal psoas muscle thickness (TPMT) <12 mm/m and <8 mm/m in men and women, respectively, was used to define sarcopenia.

The overall cohort of the published manuscripts included 2036 patients (Supplemental Table S2, http://links.lww.com/HC9/C162), of whom 940 patients were reported with compensated cirrhosis. Two authors reported including patients with previous decompensation, but compensated at inclusion.^17^^,^^19^ Concerning the cohort of compensated patients with no previous decompensation, the general characteristics are summarized in Table 1. The cohort included 829 patients. Male sex was more prevalent (39%–72%),^19^^,^^20^ with a median age of 57–66 years old. The etiologies, including alcohol-related, viral-related, and metabolic-related liver disease, ranged between 4% and 38%,^20^^,^^21^ 38% and 64%^16^^,^^19^ and 9% and 30%,^14^^,^^15^^,^^17^ respectively. The median Child–Pugh and MELD scores were between 5–6 and 8–10, respectively. Prevalence of sarcopenia varies from 8% to 63%.^19^^,^^21^

**TABLE 1:** Characteristics of studies included in the systematic review referring to the cohort of patients compensated at the time of inclusion

1st Author (year)	Number of compensated patients	Age (SD)	Male sex (%)	BMI (kg/m^2^)	Main etiologies (%)	Child–Pugh score (SD)	MELD score (SD)	Median/mean measure (SD/IQR)	Prevalence of sarcopenia/ frailty (%)	Decompensation rate (%)	Outcome decompensation (HR/sHR, 95% CI)^a^	Overall mortality/LT rate (%)	Outcome mortality/LT (HR/sHR, 95% CI)
Sarcopenia													
Rodrigues et al^14^ (2019)	38	57 (8)	61%	29	ALD 34, MASLD 30, Others 36	6 (2)	8 (3)	46.4 (40.9–52.3)	45%	47%	0.97 (0.89–1.05)	34%	0.66 (0.12–3.62)
Tapper et al^15^ (2019)	110	57 (11)	62%	29	MASLD 30, ALD 10, Viral 40, Others 20	5 (1)	8 (2)	32.2 (13.7–61.5)	—	21%	1.06 (0.91–1.25)	11%	1.004 (0.996–1.011)
Beer et al^16^ (2020)	110	58 (14)	59%	25	Viral 38, ALD 11, Others 51	—	—	12.578 (12.36–13.69)	18%	18%	1.41 (0.46–4.25)	21%	—
Ishizu et al^17^ (2021)	169	67 (10)	47%	23	Viral 47, ALD 15, MASLD 9, Others 29	5 (1)	8 (2)	43.0 (38.0–49.5)	—	28%	0.79 (0.42–1.50)	12%	0.79 (0.29–2.16)
Paternostro et al^18^ (2021)	54	55 (14)	68%	26	ALD 28, Viral 43, MASLD 13, Others 16	—	10 (3)	9.48	24%	41%	3.88 (0.90–16.50)	17%	2.40 (0.59–9.57)
Dajti et al^19^ (2022)	144	67 (14)	72%	26	Viral 64, MASLD 30, ALD 5, Others 1	5 (1)	9 (1)	44.6 (36.7–51.3)	63%	10%	2.81 (0.72–11.10)	10%	3.04 (0.89–10.42)
Luo et al^20^ (2023)	46	57 (15)	39%	25	ALD 4, MASLD 22, Viral 24, Others 50	6 (1)	9 (3)	42.6 (37.8–46.7)	—	11%	4.98 (0.35–70.78)	4%	13.61 (0.90–1000)
Di Cola et al^21^ (2024)	158	63 (10)	68%	26	Viral 44, ALD 38, MASLD 11, Others 7	5 (1)	9 (2)	51 (48.7–51.6)	8%	19%	1.29 (0.63–2.62)	4%	1.24 (0.23–6.80)
Frailty													
Kremer et al^22^ (2020)	87	61 (6)	57%	29	ALD 9	—	9 (3)	3 ± 2		11%	Frail: 8.50 (1.07–67.32)	13%	Frail: 7.66 (0.98–59.98)
Siramolpiwat et al^23^ (2021)	112	62 (9)	54%		—	6 (1)	9 (3)	3.9 ± 0.79	26%	10%	Frail: 3.01 (1.04–8.68)	4%	Frail: 2.08 (0.76–5.7)
Wang et al^7^ (2021)	—	—	—	—	—	—	—	—	—	35%	—	14%	—
Luo et al^20^ (2023)	57	57 (18)	42%	25	ALD 6, MALSD 21, Viral 19, Others 54	6 (1)	9 (3)	—	—	11%	2.66 (0.25–28.04)	—	—

^a^ Estimates expressed as subhazard ratios using liver transplantation (Ishizu et al and Paternostro et al) or liver transplantation and death (Dajti et al, Luo et al, and Di Cola et al) as competing risks.

Abbreviations: ALD, alcohol-associated liver disease; LT, liver transplant; MASLD, metabolic dysfunction–associated steatotic liver disease.

#### Association between sarcopenia and outcomes: decompensation and mortality

Three studies^14^^–^^16^ used Cox regression analysis (risk reported as hazard ratio), and the remaining 5 studies used Fine and Gray competitive risk analysis (risk reported as subhazard ratio). For the outcome of decompensation, the competing events were liver transplantation in 2 studies^17^^,^^18^ and liver transplantation or death in 3 studies.^19^^–^^21^ For the outcome of mortality, the competing event was liver transplantation in 4 studies^17^^–^^19^^,^^21^ and liver transplantation or non-liver-related death for 1 study.^20^ The final multivariable models most commonly included age, sex, parameters reflecting liver function (MELD, Child–Pugh score, albumin), and severity of portal hypertension (liver stiffness, HVPG), but there was marked heterogeneity among studies, as reported in Table 1. In the original cohorts, 3 studies^18^^–^^20^ found a significant association between sarcopenia and risk of decompensation, and 4 studies^16^^,^^18^^–^^20^ found a significant association between sarcopenia and mortality (Supplemental Table S2, http://links.lww.com/HC9/C162). In the cohort of patients with only compensated cirrhosis, none of the studies found a significant association between sarcopenia and decompensation or mortality.

### Frailty

#### Characteristics of included studies and prevalence of frailty

The general characteristics of the included studies are summarized in (Supplemental Table S2, http://links.lww.com/HC9/C162). Four prospective studies of frailty were conducted in Asia (2), Europe, and North America. They considered decompensation to be clinically detected ascites, variceal bleeding, and HE leading to hospitalization. Only 1 study considered infection as a decompensating event.^20^ Frailty was assessed using the Liver Frailty Index (LFI)^25^ in 2 out of 3 studies, whereas in the other studies, the Clinical Frailty Scale^22^ and the Fried Frailty Phenotype^20^ were used. Overall, 1506 patients were included, with 552 compensated patients. The mean age ranged from 55 to 63 years,^22^^,^^23^ with a higher prevalence in male patients (52%–77%).^7^^,^^20^^,^^22^^,^^23^ The prevalence of frailty varies from 1.4% to 38%.^20^^,^^22^ Patients compensated at inclusion, with no previous episode of decompensation (n=256), the data are summarized in Table 1. In 1 study,^7^ it was not possible to retrieve the data concerning this specific population.

#### Association between frailty, decompensation, and mortality

Given the small number of studies and heterogeneous methods of assessment, a meta-analysis was not performed. The overall decompensation rate in the entire cohort varied from 10.5% to 35%,^7^^,^^20^ whereas the mortality rate ranged from 3.9% to 14.3%^7^^,^^23^ (Table 1). When the 256 compensated cirrhosis patients without previous decompensation were assessed, available in 3 studies, the decompensation rate ranged from 9% to 10.5%,^20^^,^^22^^,^^23^ and the mortality rate from 5.2% to 5.8%.^22^^,^^23^ Although we did not conduct a meta-analysis, 2 studies^22^^,^^23^ found an association between frailty and decompensation [aHR ranging from 3.01 (1.04–8.68) to 8.50 (1.07–67.32)] and death [aHR ranging from 2.08 (0.76–5.70) to 7.66 (0.98–59.98)], suggesting that frailty is a negative predictive factor in patients with compensated cirrhosis.

## DISCUSSION

This systematic review included 8 studies on sarcopenia and 4 studies on frailty in patients with compensated cirrhosis. We evaluated the association of these conditions with decompensation and, as a secondary outcome, mortality, which is of clinical importance and represents one of the key open questions of the Baveno VII research agenda.^9^ While data regarding the prognostic role of frailty and sarcopenia in decompensated disease are well-established,^4^^,^^25^^–^^27^ data on compensated diseases are scant and pose additional questions about the cause-and-effect relationship between sarcopenia and the natural history of compensated cirrhosis. Interestingly, the overall prevalence of sarcopenia in our study population with compensated cirrhosis was 47%, ranging from 29% to 65%. In a recent meta-analysis, among LT candidates, mostly decompensated, the prevalence of sarcopenia was 46%.^26^ Our results suggest that sarcopenia frequently remains undiagnosed in patients with compensated cirrhosis.

We systematically assessed for the first time the prognostic impact of sarcopenia in a well-characterized cohort of compensated cirrhosis without any episode of previous decompensation. We observed considerable variability in outcome definitions, analytical methods, and the variables incorporated into the multivariable models. The definition of compensated cirrhosis was not homogeneous, with some cohorts including patients who had recovered from decompensation over a year before. As such, we analyzed the data a step further, asking the authors to clarify their definition of compensated cirrhosis. We found that the risk of decompensation related to sarcopenia was not increased in the majority of the studies evaluated with a population of patients with compensated cirrhosis according to Baveno VII.^9^ Similarly, sarcopenia was not independently associated with an increased risk of death in most of the included studies. The follow-up period varied substantially, ranging from 16 to 61 months, with only half of the studies including a follow-up duration of at least 3 years. This limited timeframe in some studies may have been insufficient to capture clinical events.

Our review highlights several important areas that future prospective studies should address in the field of compensated cirrhosis and sarcopenia. One key issue is patient selection—whether all patients with cirrhosis should be screened for sarcopenia from the start, or should screening focus only on those with more advanced compensated disease. In the studies we reviewed, more “advanced” compensated patients often included those with Child–Pugh A6 or B7 scores, low albumin levels, clinically significant portal hypertension (CSPH), and sometimes even a history of decompensation. It seems that the latter group has a different natural history with a higher rate of liver-related events.^10^ In order to better understand how sarcopenia relates to different stages of compensated disease, we believe a large multicenter prospective study is needed, focusing specifically on patients who have never decompensated. This study should include a well-balanced group of patients, for age and sex, as well as the prevalence of patients with and without signs of CSPH, to properly assess the first decompensation. In addition, the follow-up time should be a minimum of 3 years to allow time for patients to develop events.

An additional key consideration is the method used to assess sarcopenia. CT-based analysis at the L3 vertebra is currently the gold standard for measuring muscle mass and is recommended by EASL (European Association for the Study of the Liver) nutrition guidelines.^27^ It has been shown to predict mortality,^26^ especially in decompensated patients. However, it has not been widely studied as a predictor of decompensation. Even so, CT remains the most accurate and widely available method for assessing sarcopenia. In our review, most studies included L3 in CT, but one included MRI^16^ and another selected another vertebral level, T12.^15^ Although we suppose that other areas and imaging methods could provide prognostic data in patients with cirrhosis,^15^ L3 with CT remains the most widely explored and validated. Its downsides, radiation exposure and cost, make it less ideal for repeated measurements; however, low-radiation CT may be a better option in the future. For now, CT imaging is a practical and reliable tool for baseline sarcopenia assessment in prospective studies.

Among the various frailty assessment tools, handgrip strength^28^ and the Liver Frailty Index^25^ emerged as the methods with the highest levels of supporting evidence. Their predictive value for clinical outcomes reinforces the importance of incorporating standardized frailty assessments into the routine evaluation and risk stratification of this patient population. In our review, 2 studies used other scales that were not developed in patients with liver cirrhosis; thus, their impact has been less validated in this setting.

Notably, our study highlights the high prevalence in compensated patients, who are not routinely subjected to diagnostic measures to assess sarcopenia and frailty. Thus, interventions to mitigate them are often not applied, as in more advanced stages of liver disease. One could hypothesize that if diagnosed and treated during a compensated stage, one could potentially prevent a first decompensating event. A recent study showed that very slight changes in ΔLFI at 3 months were associated with a clinically meaningful reduction in waitlist mortality,^29^ demonstrating that minor improvements can have an impact. Although sarcopenia can be driven by endotoxemia, lower testosterone levels, and hyperammonemia,^30^^,^^31^ much more common in decompensated disease, and not routinely detected in compensated cirrhosis, observational studies support the role of sarcopenia in developing decompensation and worsening prognosis, even in the early phase of compensated disease.^18^^,^^19^ The exact mechanism or drivers behind the development of sarcopenia in the early stages of cirrhosis remain unclear. While our data cannot prove a causal relationship between sarcopenia and frailty and decompensation, some studies found an association with risk for first decompensation and found that sarcopenia can be prevalent in compensated cirrhosis. In a study conducted with ACLD patients stratified according to Child–Pugh class, MELD score, and HVPG, the authors concluded that sarcopenia was associated with increased mortality at any stage of liver disease. Besides sarcopenia, there are other aspects of body composition, such as bone, muscle, and fat density, which have also shown good performance in predicting outcomes even in the initial stages of liver disease, significantly outperforming MELD.^15^^,^^21^

Concerning frailty, despite scarcer data, our review suggests that frailty can negatively affect outcomes in compensated cirrhosis, prompting the early recognition of sarcopenia and frailty, even at this disease stage.

This study had some important limitations. First, the restrictive inclusion criteria implied that a small number of studies were considered eligible for the analysis, and even these included highly heterogeneous patient groups and analyses performed, limiting the possibility of performing a meta-analysis. However, this also reflects the lack of data in the literature regarding sarcopenia and frailty in this specific population of patients with compensated cirrhosis. Second, the definition of compensated cirrhosis also varies among studies. Whereas some considered compensated cirrhosis patients to be those who did not have a first decompensating event, others included patients who had experienced variceal bleeding or ascites in the past, but at the time of inclusion did not have signs of decompensation. The authors did not specify whether these latter patients fulfilled the recently proposed criteria for recompensation.^9^ Nonetheless, the variables: age, gender, and liver function were constantly included in the multivariable analysis. In addition, we did not control for comorbidities independently associated with sarcopenia (heart or kidney disease), the presence of clinically significant portal hypertension, or therapies that prevent decompensation, namely, beta-blockers.^32^^,^^33^

The strengths of our study include its novelty, as it is the first study to systematically review and synthesize the prognostic relevance of sarcopenia in the compensated stage of ACLD. We reported that in this cohort, sarcopenia was prevalent, but did not lead to an independent, increased risk of decompensation and death in all studies. Our review highlights the small number of published cohorts solely with compensated cirrhosis, non-uniform definitions of compensated disease and lack of granularity to distinguish those in a more advanced compensated stage, in addition to unstandardized sarcopenia/frailty assessment methods. In response, the authors have initiated a large, multicenter, prospective study within the Baveno Cooperation, with a clear definition of compensated cirrhosis, an adequately long follow-up period, and a consistent and well-defined screening method to assess adequately the impact of body composition and functional changes on the natural history of compensated cirrhosis.

## Supplementary Material
