# Supplementary material for: The impact of sarcopenia and frailty on decompensation in compensated cirrhosis: A systematic review
Source: Hepatol Commun. 2025 Oct 21;9(11):e0811. doi: 10.1097/HC9.0000000000000811 (PMC12548989; doi:10.1097/HC9.0000000000000811)
Supplement: Supplementary file 1 [file hc9-9-e0811-s001.docx]

**SUPPLEMENTARY MATERIAL**

**Search strategy**

**Sarcopenia**

- **PubMed:** ("Sarcopenia"[Mesh] OR sarcopenia[tiab] OR "muscle mass loss"[tiab] OR "muscle wasting"[tiab] OR "muscle atrophy"[tiab] OR "muscle degeneration"[tiab]) AND ("Liver Diseases"[Mesh] OR "Liver Cirrhosis"[Mesh] OR "chronic liver disease"[tiab] OR "hepatic disease"[tiab] OR cirrhosis[tiab] OR "liver fibrosis"[tiab]) AND ("journal article"[Publication Type]) NOT ("review"[Publication Type] OR "systematic review"[Publication Type] OR "meta-analysis"[Publication Type]).
- **EMBASE:** ('sarcopenia'/exp OR sarcopenia:ti,ab OR 'muscle mass loss':ti,ab OR 'muscle wasting':ti,ab OR 'muscle atrophy':ti,ab) AND ('liver disease'/exp OR 'liver cirrhosis'/exp OR 'chronic liver disease':ti,ab OR 'hepatic disease':ti,ab OR cirrhosis:ti,ab OR 'liver fibrosis':ti,ab) AND [article]/lim NOT ('review'/it OR 'systematic review'/it OR 'meta analysis'/it)

**Frailty**

- **PubMed:** ("Frailty"[MeSH Terms] OR frailty[tiab] OR "physical frailty"[tiab] OR "frailty syndrome"[tiab]) AND ("Liver Diseases"[MeSH Terms] OR "Liver Cirrhosis"[MeSH Terms] OR "chronic liver disease"[tiab] OR "hepatic disease"[tiab] OR cirrhosis[tiab] OR "liver fibrosis"[tiab]) AND ("journal article"[Publication Type]) NOT ("review"[Publication Type] OR "systematic review"[Publication Type] OR "meta-analysis"[Publication Type])
- **EMBASE:** ('frailty'/exp OR frailty:ti,ab OR 'physical frailty':ti,ab OR 'frailty syndrome':ti,ab) AND ('liver disease'/exp OR 'liver cirrhosis'/exp OR 'chronic liver disease':ti,ab OR 'hepatic disease':ti,ab OR cirrhosis:ti,ab OR 'liver fibrosis':ti,ab) AND

[article]/lim NOT ([review]/lim OR 'systematic review':it OR 'meta analysis':it)

**Supp. Table 1** - Sarcopenia and Frailty in Compensated Cirrhosis Systematic Review – Newcastle Ottawa Scale.

| **Author/**  **Year** | **Selection** | | | | **Comparability** | **Outcome** | | | **Study design**  **(1 point if prospective)** | **Total (max. 9)** |
| --- | --- | --- | --- | --- | --- | --- | --- | --- | --- | --- |
|  | Representativeness of exposed cohort | Selection of the non-exposed cohort | Ascertainment of exposure (in medical records) | Demonstration outcome not present at start of study | Comparability of cohorts on the basis of the design or analysis | Assessment of outcome | Was follow-up long enough for outcomes to occur | Adequacy of follow-up |  |  |
| **Sarcopenia** | | | | | | | | | | |
| **Beer (2020)** | * | * | * | * | * | * | * | * |  | 8 |
| **Rodrigues (2019)** | * | * | * | * | * | * |  | * | * | 8 |
| **Tapper (2019)** | * | * | * | * |  | * | * | * |  | 7 |
| **Ishizu (2021)** | * | * | * | * |  | * | * | * |  | 7 |
| **Paternostro (2021)** | * | * | * | * | * | * | * | * |  | 8 |
| **Colecchia (2022)** | * | * | * | * | * | * | * | * |  | 8 |
| **Luo**  **(2023)** | * | * | * | * | * | * |  | * | * | 8 |
| **Di Cola**  **(2024)** | * | * | * | * | * | * | * | * | * | 9 |
| **Frailty** | | | | | | | | | | |
| **Siramolpiwat (2021)** | * | * | * | * | * | * |  | * | * | 8 |
| **Wang (2021)** | * | * | * | * | * | * | * | * | * | 9 |
| **Kremer (2020)** | * | * | * | * | * | * |  | * | * | 8 |
| **Luo**  **(2023)** | * | * | * | * | * | * |  | * | * | 8 |
