# Supplementary material for: The impact of sarcopenia and frailty on decompensation in compensated cirrhosis: A systematic review
Source: Hepatol Commun. 2025 Oct 21;9(11):e0811. doi: 10.1097/HC9.0000000000000811 (PMC12548989; doi:10.1097/HC9.0000000000000811)
Supplement: Supplementary file 2 [file hc9-9-e0811-s002.docx]

**TABLES**

**Table 1** – Characteristics of studies comprising the overall cohort included in the study for sarcopenia and frailty.

| **1^st^ Author, (Year)** | **Country** | **Design** | **Nr of patients overall (compensated)** | **Age (SD)** | **Male sex (%)** | **BMI (kg/m^2^)** | **Main aetiologies (%)** | **Child Pugh score (SD)** | **MELD score (SD)** | **Main Sarcopenia/ frailty definition** | **Main cut-off**  **(M, F)**** | **Median/**  **mean measure (SD/IQR)** | **Prevalence of sarcopenia/ frailty (%)** | **Follow-up**  **(mo.)** | **Statistical approach°** | **Variables in the MV analysis** | **Decompensation rate (%)** | **Outcome decompensation (HR/sHR, 95% CI)°** | **Overall mortality/LT rate (%)** | **Outcome mortality/LT**  **(HR/sHR, 95% CI)°** |
| --- | --- | --- | --- | --- | --- | --- | --- | --- | --- | --- | --- | --- | --- | --- | --- | --- | --- | --- | --- | --- |
| **Sarcopenia** |  |  |  |  |  |  |  |  |  |  |  |  |  |  |  |  |  |  |  |  |
| **Rodrigues et al.^20^ (2019)** | Switzerland | Prosp | 84 (38) | 57 (11) | 61 | 28 | ALD 51, MASLD 24,  Others 25 | 7 (2) | 13 (8) | SMI at L3  CT scan | <50, <39 | 44 | 59% | 12 | Cox Regression (HR) | Liver stifness, platelet count, total adipose tissue index, visceral adipose tissue index; subcutaneous adipose tissue index for decompensation.  MELD-Na and intramuscular adipose tissue index for mortality | 43 (51%) | 1.21 (0.59-2.48) | 16 (19%) | 1.09 (0.39-3.06) |
| **Tapper et al.^14^ (2019)** | USA | Retro | 274 (110) | 58 (5) | 57 | 29 | ALD 21, MASLD 32, Viral 29, Others 18 | 7 (2) | 11 (5) | Dorsal muscle group at T12  CT scan | - | - | - | 61 | Cox Regression (HR) | Features of body composition and MELD for mortality and decompensation | - | - | 114 (43%) | 1.004 (0.997–1.011) |
| **Beer et al.^18^**  **(2020)** | Austria | Retro | 265 (110) | 57 (13) | 66 | 25 | Viral 32, ALD 23, Others 45 | 6 | 11 (8) | TPMT at L3 MRI scan | <12, <8 | 12.2 (12.0-12.9) | 28% | 30 | Cox Regression (HR) | Sarcopenia, MELD, albumin varices and CSPH for mortality and decompensation | 73 (35%) | 1.34 (0.77-2.33) | 67 (32%) | 2.16 (1.29-3.62) |
| **Ishizu et al.^15^**  **(2021)** | Japan | Retro | 335 (215) | 63 (7) | 52 | 23 | ALD 21, Viral 44, Others 35 | 6 (2) | 10 (4) | SMI at L3  CT scan | <42, <38 | 43.7 (38-50) | - | 43 | Fine and Gray competitive risk analysis (sHR) | Age, sex, etiology, Child Pugh, visceral fat, sarcopenia for mortality and decompensation | 25 (10%) | 0.89 (0.50 - 1.51) | 81 (25%) | 1.16 (0.74 - 1.83) |
| **Paternostro et al.^17^ (2021)** | Austria | Retro | 203 (54) | 55 (11) | 68 | 25 | ALD 54, Viral 25, MASLD 7, Others 14 | - | 12 (4) | TPMT at L3  CT scan | <12, <8 | 12 ± 3.4 | 38% | 27 | Fine and Gray competitive risk analysis (sHR) | Age, sex, BMI, sarcopenia, HVPG, albumin for mortality and decompensation | 108 (53%) | 1.79 (1.23-2.61) | 69 (34%) | 2.23 (1.39-3.56) |
| **Dajti et al.^19^ (2022)** | Italy | Retro | 209 (209) | 66 (9) | 73 | 25 | Viral 58, MASLD 32, ALD 4, Others 6 | 5 (1) | 9 (2) | SMI at L3  CT scan | <50, <39 | 43 (37-51) | 64% | 37 | Fine and Gray competitive risk analysis (sHR) | Liver stiffness, spontaneous portosystemic shunts, sarcopenia for mortality and decompensation | 52 (33%) | 2.08 (1.09–3.99) | 30 (14%) | 2.74 (1.11-6.82) |
| **Luo et al.^16^ (2023)** | China | Prosp | 233 (46) | 61  (15) | 51 | 25 | Viral 23, ALD 19, MASLD 14,  Others 45 | 7 (3) | 12 (6) | SMI at L3  CT scan | <45, <33 | 42 (37-48) | 30% | 20 | Fine and Gray competitive risk analysis (sHR) | Age, sex, cirrhosis severity and comorbidities for mortality and decompensation | 94 (40%) | 2.03 (1.31-3.14) | 41 (18%) | 2.28 (1.19-4.36) |
| **Di Cola et al.^21^ (2024)** | Italy | Prosp | 433 (158) | 57 (9) | 71 | 28 | ALD 40, Viral 29,  MASLD 15, Others 16 | 8 (2) | 13 (5) | SMI at L3  CT scan | <50, <39 | 50 (48-52) | 39% | 12 | Fine and Gray competitive risk analysis (sHR) | Muscle changes, MELD, ascites, over hepatic encephalopathy for mortality and decompensation | 143 (31%) | 1.19 (0.89 – 1.6) | 51 (12%) | 1.38 (0.81-2.37) |
| **Frailty** |  |  |  |  |  |  |  |  |  |  |  |  |  |  |  |  |  |  |  |  |
| **Kremer et al.^24^ (2020)** | Germany | Prosp | 299 (140) | 63 (9) | 57 | 26 | ALD 24 | - | 9 (3) | Clinical Frailty Score | >4 | 3 (2-3) | Frail 1% Pre-frail 6% | 13 | Cox Regression (HR) | MELD, hepatic encephalopathy, Clinical Frailty Score, albumin | - | - | 39 (13%) | 1.53 (1.12–2.10) |
| **Siramolpiwat et al.^25^ (2021)** | Thailand | Prosp | 152 (152) | 55 (11) | 70 | 25 | ALD 28, Viral 54, Others 18 | - | 9 (3) | Liver frailty index | >4.4 | 3.9 | Frail 25 % | 15 | Cox Regression (HR | Albumin, bilirubin, natrium, MELD, MELD Na, Child Pugh B, frailty for decompensation | 52 (10%) | Frail: 3.01 (1.04-8.68) | 6 (4%) | Frail: 2.08 (0.76–5.7) |
| **Wang et al.^7^ (2021)** | North America/ India | Prosp | 822 (203) | 60 (6) | 77 | 28 | Viral 26,  ALD 20,  MASLD 23,  Others 31 | - | 9 (3) | Liver frailty index | >4.4 | 3.66 | Frail 11 % | 51 | Fine and Gray competitive risk analysis (sHR) | Age, sex, MELD, frailty for mortality and decompensation | 60 (7%) | 2.85 (1.94–4.19) | 187 (23%) | 3.97; (2.26–6.97) |
| **Luo et al.^16^ (2023)** | China | Prosp | 233 (57) | 61  (15) | 52 | 25 | Viral 23, ALD 20, MASLD 13,  Others 44 | 7 (3) | 12 (6) | Fried Frailty Phenotype | ≥3 | - | Frail 38% | 20 | Fine and Gray competitive risk analysis (sHR) | Muscle changes, MELD, ascites, over hepatic encephalopathy for mortality and decompensation | 105 (38%) | 2.12 (1.37-3.27) | 47 (17%) | 2.23 (1.08-4.59) |

***Unit for SMI and PMI: cm^2^/m^2^, TPMT: mm*

**Abbreviations:** ALD: alcohol-related liver disease; BMI: body mass index; CSPH: clinical significant portal hypertension; CT: computed tomography; HR: hazard ratio; LT: liver transplant; MASLD: metabolic dysfunction-associated steatotic liver disease; MELD: model for End Stage Liver Disease; MRI: magnetic resonance imaging; MV: multivariable; PMI: psoas muscle index; Prosp: prospective; Retro: retrospective; SD: standard deviation; sHR: sub-hazard ratio; SMI: skeletal muscle index; TPMT: transversal psoas muscle thickness.

*°Estimates expressed as subhazard ratios using liver transplantation (Ishizu et al, Paternostro et al.) or liver transplantation and death (Dajti et al, Luo et al, Di Cola et al) as competing risks*
